# Homozygous Carriers of F2 c.20210G>A Variant: A Report of Two Cases and Literature Review

**DOI:** 10.7759/cureus.36668

**Published:** 2023-03-25

**Authors:** Caitlin M Raymond, Duc-Hieu Bui, Jianli Dong

**Affiliations:** 1 Pathology, University of Texas Medical Branch, Galveston, USA; 2 Medicine, John Sealy School of Medicine, University of Texas Medical Branch, Galveston, USA

**Keywords:** phenotypic heterogeneity, genetic variant, thrombophilia, heterozygous, homozygous, factor v leiden, f2 c.20210g>a, genetic and environmental risk factors, coagulation factor v gene f5, coagulation factor ii (thrombin) gene f2

## Abstract

Thromboembolism is known to be a multifactorial event that is impacted by various genetic and environmental factors. The genetics society's recommended name for this variant is c.*97G>A (this is the nomenclature we need to use in the patient report). However, people have been using legacy names c.20210G>A or G20210A (so these are common names). One of the most common genetic variants associated with inherited thrombophilias, F2 c.20210G>A is acknowledged to be a weak but significant risk factor for thromboembolism. However, its clinical presentation has been described as phenotypically heterogeneous. We present two rare cases with homozygous F2 c.20210G>A variant, one of which also carries a heterozygous variant in coagulation factor V gene F5, c.1601G>A (p.Arg534Gln; commonly known as factor V Leiden). We described the clinical courses of these two cases and discussed F2 c.20210G>A and factor V Leiden as genetic risk factors in thromboembolism, the role of provoking factors, such as surgery and malignancy, and the management of such patients.

## Introduction

Coagulation factor II, also known as thrombin, is encoded by the F2 gene. It is a vitamin K-dependent coagulation factor and has enzymatic activity that converts fibrinogen to fibrin allowing for the development of a fibrin clot. An inappropriately high level of fibrin clot development, also known as thromboembolism or venous embolism, can lead to serious health problems including pulmonary embolism, stroke, and myocardial infarction. Several genetic variants in F2 have been described, resulting in either gain of function and increased thrombosis [1], or loss of function and increased bleeding [2]. One of the most prevalent in the population is the c.*97G>A variant, commonly known as c.20210G>A or G20210A, which is present in approximately 18% of patients of European ancestry who experience venous blood clots, which results in substitution of an adenine for a guanine in the 3’ untranslated region of the gene, leading to improved processing of mRNA transcript. This results in increased levels of F2 mRNA and subsequently thrombin protein which is most notable in homozygous carriers [3,4]. Increased levels of thrombin protein result in increased enzymatic processing of fibrinogen to fibrin, with increased fibrin clot formation. This can lead to thromboembolism, particularly occurring in the veins, with increased risk of life-threatening events such as pulmonary embolism and stroke. Thus, F2 c.20210G>A acts as a gain of function variant and has been observed to be associated with an increased risk of both venous embolism and arterial thrombosis [4,5].

The prevalence of this variant has been found to vary widely based on ethnicity; however, most studies show that the prevalence is highest in patients of European descent (1.2-4.6%) and in patients of Jewish descent (1-6.7%) [3-5]. The vast majority of c.20210G>A carriers are found as heterozygous, with homozygous being acknowledged to be quite rare, and only approximately 100 cases reported in the literature since its discovery in 1996 [6].

Studies on the rare cases carrying homozygous F2 c.20210G>A have indicated a higher risk of thromboembolism than heterozygous, although the exact risk ratio remains difficult to determine. This is due in part to the marked phenotypic heterogeneity of known homozygous cases, meaning patients' presentation can vary widely when carrying this genotype [3]. Presentations range from multiple severe embolisms in the neonatal period to asymptomatic despite multiple provoking risk factors, such as surgery and malignancy. The current consensus is that F2 c.20210G>A variant is a weak, but significant, risk factor for thromboembolism and interacts with multiple inherited and acquired factors to affect phenotypic expression. In this report, we present two cases of homozygous F2 c.20210G>A carriers, which represent wide range of phenotypes seen. In the review of these cases, we discussed the role of provoking factors such as surgery and malignancy and the management of such patients.

## Case presentation

Case 1

A 33-year-old Caucasian male, non-smoker, with no significant past medical history was found to have medulloblastoma of the cerebellum after experiencing progressive nausea, vomiting, and dizziness. The patient was treated with surgical resection with post-operative radiation therapy. Three months into radiation therapy, the patient developed shortness of breath and pleuritic chest pain. Upon presentation to the emergency department, he was found to have a pulmonary embolism (PE). The patient had no prior history of thromboembolism and no family history of a clotting disorder.

A hypercoagulability workup was performed, and the patient was found to be negative for antiphospholipid antibody syndrome with unremarkable results for prothrombin time (PT)/international normalized ratio (INR) and activated partial thromboplastin time (aPTT). However, molecular diagnostics uncovered both a homozygous F2 variant - NM_000506.5(F2):c.*97G>A (commonly known as c.20210G>A or G20210A) and a heterozygous factor V Leiden - NM_000130.4(F5):c.1601G>A (p.Arg534Gln). The patient was placed on a direct-acting oral anticoagulant (DOAC) indefinitely. Recurrence of disease occurred three years later, and surgical resection was performed. Seven months post-resection, the patient was admitted with obstructive hydrocephalus that was treated successfully with placement of an intraventricular shunt. Subsequent surveillance imaging revealed continued tumor progression and palliative care was discussed. The patient was discharged for home health and passed away shortly after. No thrombotic events occurred since DOAC therapy was initiated.

Case 2

A 74-year-old Caucasian female, non-smoker, with a history of recurrent PE and deep venous thrombosis (DVT) was referred to the anticoagulation clinic for management of chronic warfarin use. She reported the onset of blood clots at the age of 16 years with over 14 separate instances since then. The patient has been on enoxaparin or warfarin since adolescence, but for the last 10 years, has only been taking warfarin. She was pregnant three times with three full-term live births and no pregnancy complications. The patient also notes the last instance of PE occurring during a two-three-year period in which she had discontinued warfarin for reasons unknown. Her son has also been reported to have a history of unprovoked PE and is being treated with DOAC.

A hypercoagulability workup was performed, and the patient was noted to be negative for antiphospholipid antibodies and lupus anticoagulant. Coagulation parameters were unremarkable, although fibrinogen levels were elevated. Molecular diagnostics showed negative results for factor V Leiden but uncovered a homozygous F2 c.20210G>A variant. The patient was ultimately placed on a DOAC indefinitely. Since then, she has had a number of provoking events, namely a hip and humerus fracture with subsequent corrective surgical procedures. Yet, there have been no thromboembolic occurrences during the four-year follow-up after her F2 test.

## Discussion

**Figure 1:**
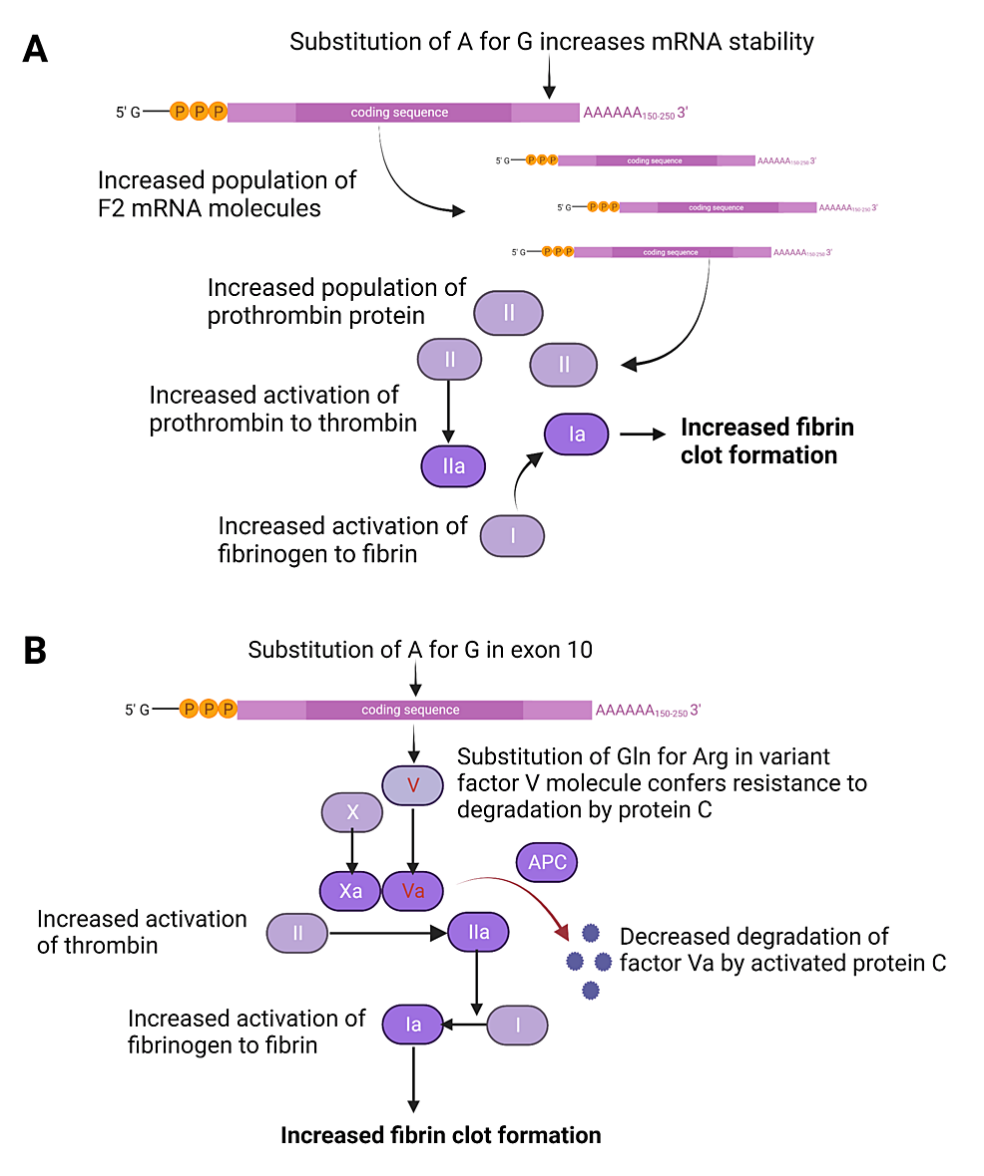
Mechanism of action of common F2 and F5 variants. The images show (A) the F2 c.20210G>A variant results in a substitution of adenine for guanine in the 3’ untranslated region of the mRNA transcript. This results in increased mRNA processing and stability and leads to increased levels of F2 protein (coagulation factor II prothrombin). When activated to thrombin, F2 protein converts fibrinogen to fibrin, ultimately resulting in increased fibrin clot formation. (B) The F5 Leiden variant results in a substitution of adenine for guanine in exon 10 of the F5 gene. This results in substitution of glutamine for arginine in the variant protein and confers resistance to degradation by activated protein C. Activated factor V complexes with activated factor X convert prothrombin to thrombin, ultimately leading to increased fibrin clot formation. A: adenine; G: guanine; mRNA: messenger RNA; F2: factor II gene; II: prothrombin protein; IIa: activated prothrombin protein (or thrombin); I: fibrinogen; Ia: activated fibrinogen (fibrin); X: protein factor X; Xa: activated factor X; V: factor V protein; Va: activated factor V protein; APC: activated protein C The image is created by the author (Caitlin Raymond) of this study with the help of BioRender.com.

The F2 c.20210G>A variant acts to stabilize F2 mRNA, ultimately resulting in more thrombin protein (Figure 1A). While acknowledged to be a weak, but significant, risk factor for thromboembolism, homozygous F2 c.20210G>A carriers have been observed to have a large spectrum of presentations [3]. Our first case is remarkable for the presence of both a homozygous F2 c.20210G>A as well as a heterozygous factor V Leiden variant. The F5 c.1601G>A (p.Arg534Gln) is a single nucleotide substitution in exon 10 that confers resistance of the variant factor V protein to degradation by activated protein C (Figure 1B). The presence of both these genetic variations is widely recognized to have a compound effect and has been shown to increase the relative risk of embolism. However, most of the studies on these common F2 and F5 variants were performed on heterozygous carriers [7,8]. The genotype of one of our patients - homozygous for F2 c.20210G>A plus heterozygous for factor V Leiden - is estimated to occur in only one in 200,000 persons worldwide [9].

To our knowledge, there are only a limited number of reported cases of patients carrying homozygous F2 c.20210G>A variant, and the majority of these cases presented with unprovoked thromboembolism [8-12]. Our first case is unique in that the patient had no history of thromboembolism or family history of thromboembolism until his presentation with PE following surgery for malignancy. Undoubtedly, malignancy and the associated surgery were provoking factors in this patient, and the discovery of his concurrent risk factors of homozygous F2 variant as well as heterozygous factor V Leiden were unexpected. In contrast, our second case featured a more severe clinical presentation, with thromboembolic events from the age of 16 including both DVTs and PEs. This patient required lifelong treatment with anticoagulants to prevent severe outcomes, although her homozygous F2 c.20210G>A status was not discovered until her seventh decade. Arguably, our second patient had less genetic predisposition to thromboembolism than our first, who also carried a heterozygous factor V Leiden variant. While advancing age is a risk factor for thromboembolism, it is important to note that our second case experienced thromboembolism from her adolescence, when the general risk of thromboembolism is known to be relatively low [10]. It is also interesting to consider the differences in gender between the two cases. While there is limited research into the role of gender in thromboembolism, studies have found no difference in absolute risk of venous embolism between men and women, outside of risk factors unique to women such as pregnancy, the post-partum period, oral contraceptive therapy, and hormone replacement therapy [10].

Our second case is a compelling example for discussing the management of thrombophilia, especially in homozygous F2 c.20210G>A carriers. Over the course of her long life, this patient was treated with a variety of anticoagulants, including injectable low molecular weight heparin, oral warfarin tablets, and ultimately DOAC. There are currently no specific guidelines for the management of inherited thrombophilia specifically due to homozygous F2 c.20210G>A, although DOACs are favored given that they often require only once-daily dosing, have limited toxicity, and unlike warfarin, do not require serial monitoring to ensure dosing in the therapeutic range [12-14]. Despite this, most experts agree that long-term anticoagulation is necessary in the setting of a thromboembolic event, as well as modification of risk factors. Notable among these is the prescription of oral contraceptives. Current World Health Organization (WHO) recommendations are that oral contraceptives cannot be prescribed in women with a history of inherited thrombophilia [15]. However, a meta-analysis published in 2016 concluded that an assessment of the clinical severity of thrombophilia was necessary [15]. Patients with a family history of mild clinical manifestations of thrombophilia had only a modest increase in absolute risk of venous embolism when taking oral contraceptives (0.49-2.0 per 100-pill-years) [15].

The patient in case 2 is also notable for having three uncomplicated pregnancies delivered at term. Inherited thrombophilias are known to be associated with pregnancy complications including eclampsia/pre-eclampsia, intrauterine growth restriction, placental abruption, stillbirth, miscarriage, and pregnancy-associated venous thromboembolism [16,17]. However, given the rarity of the homozygous c.20210G>A genotype, limited research has been conducted on the associations of this inherited thrombophilia with poor pregnancy outcomes, although one study did conclude that there was an increased odds ratio of 3.76 for recurrent pregnancy loss [18]. We note that our patient was treated with anticoagulants throughout each of her pregnancies and had a personal history of recurrent venous thromboembolism.

Routine genetic testing for thrombophilia has generally not been recommended due to its inconsequential effect on treatment course, and the fact that those with inherited thrombophilia risk factors may not necessarily develop thromboembolic events [19,20]. This can lead to patients being inappropriately treated and increasing the risk of complications, such as bleeding. However, testing can be beneficial on a case-by-case basis such as those demonstrated here, as some patients may benefit from early detection and treatment of thrombophilia to prevent thromboembolic events. Screening for thrombophilia may be warranted in such cases of unprovoked or recurrent VTE, as it can help identify those that are at an increased risk. Furthermore, DOACs have emerged as effective treatments for thromboembolic events, making screening for thrombophilia even more important in identifying patients who may benefit from preventive treatment.

## Conclusions

In this report, we describe the clinical courses of two patients carrying a rare homozygous F2 c.20210G>A variant, one of which also was heterozygous for factor V Leiden. These patients had diverse clinical courses from multiple unprovoked thromboembolic events from a young age to thromboembolic events that were triggered by specific factors or events without a clear causal link to their F2 genotype. This report underscores the fact that thromboembolism is a multifactorial process, in which both genetic and environmental factors interact to increase or decrease risk of a blood clot. Management of these patients involves not only anticoagulation but also modification of risk factors.
